# Prediction of Urban Taxi Travel Demand by Using Hybrid Dynamic Graph Convolutional Network Model

**DOI:** 10.3390/s22165982

**Published:** 2022-08-10

**Authors:** Jinbao Zhao, Weichao Kong, Meng Zhou, Tianwei Zhou, Yuejuan Xu, Mingxing Li

**Affiliations:** 1 School of Transportation and Vehicle Engineering, Shandong University of Technology, Zibo 255000, China; 2 School of Transportation, Southeast University, Nanjing 211189, China

**Keywords:** travel demand prediction, spatial-temporal model, hybrid dynamic graph convolutional network, deep learning

## Abstract

The efficient and accurate prediction of urban travel demand, which is a hot topic in intelligent transportation research, is challenging due to its complicated spatial-temporal dependencies, dynamic nature, and uneven distribution. Most existing forecasting methods merely considered the static spatial dependencies while ignoring the influence of the diversity of dynamic demand patterns and/or uneven distribution. In this paper, we propose a traffic demand forecasting framework of a hybrid dynamic graph convolutional network (HDGCN) model to deeply capture the characteristics of urban travel demand and improve prediction accuracy. In HDGCN, traffic flow similarity graphs are designed according to the dynamic nature of travel demand, and a dynamic graph sequence is generated according to time sequence. Then, the dynamic graph convolution module and the standard graph convolution module are introduced to extract the spatial features from dynamic graphs and static graphs, respectively. Finally, the spatial features of the two components are fused and combined with the gated recurrent unit (GRU) to learn the temporal features. The efficiency and accuracy of the HDGCN model in predicting urban taxi travel demand are verified by using the taxi data from Manhattan, New York City. The modeling and comparison results demonstrate that the HDGCN model can achieve stable and effective prediction for taxi travel demand compared with the state-of-the-art baseline models. The proposed model could be used for the real-time, accurate, and efficient travel demand prediction of urban taxi and other urban transportation systems.

## 1. Introduction

Realizing the balance between travel demand and the supply of urban transportation systems is not only beneficial to improve the utilization efficiency of transportation infrastructure but also important to improve the level of service. As the demand/supply attributes of urban transportation systems in different regions vary significantly, many cities are improving their travel demand models and discussing whether the existing models are effective as well as what data are required to characterize the travel demand/supply features. On the one hand, with the development of intelligent transportation systems (ITS), more and more data from different traffic sensors, such as global positioning systems (GPS) and mobile maps, could be used to form a real-time and efficient big dataset for improving travel demand prediction. On the other hand, however, the travel demand models managed by transportation agencies have not traditionally been able to comprehensively reflect the spatial-temporal dependencies, dynamic nature, and uneven distribution of urban travel demand/supply features, thus resulting in the oversupply or undersupply of transportation systems at a specific time or area, which not only wastes public resources but also affects the residents’ travel experience. For instance, accurate demand prediction can help taxi operators and ride-hailing platforms to quickly update passengers’ travel needs and cruising locations to meet passengers’ potential demands, thus reducing passengers’ waiting time and improving the utilization rate of registered cars [1]. Therefore, using more effective and accurate models to forecast travel demand takes timely countermeasures to alleviate imbalance between supply and demand, the foci of this study.

Indeed, in terms of structuring travel demand models, researchers have made sustained effort and developed numerous models, such as the autoregressive integrated moving average (ARIMA) [2], the Kalman filtering model [3], support vector regression (SVR) [4], and recurrent neural networks RNN [5,6,7]. However, it is by no means easy to predict travel demand accurately as travel demand is not only influenced by the historical pattern and regional neighbor but is also closely linked to regions with distant but similar travel patterns [8]. Specifically, it is necessary to consider both temporal and spatial dimensions when predicting travel demand. Compared with the temporal forecasting models, relatively few methods reflect the spatial characteristics of travel demand. A common approach is to segment the study area using a grid and then apply the convolutional neural network (CNN) and its variants to construct the spatial correlations [9,10,11]. However, this approach has a natural disadvantage as it destroys the complex structure of urban road networks [1]: a residential complex or park plaza mall may be divided into two or more small units. Meanwhile, inspired by the successful application of graph convolutional networks (GCN) on non-Euclidean data, some researchers have introduced graph structures into urban traffic computation and extracted spatial correlations by using GCN [12,13,14,15].

While researchers have continued to integrate temporal feature extraction models and spatial feature extraction models into hybrid networks to improve the forecasting efficiency and accuracy [16,17,18,19], it is worth noting that the prediction performance would be less promising if only the topology of the traffic network is considered without considering the dynamic changes of the traffic situation [20]. To deal with this problem, a potentially effective method is to design a spatial-temporal forecasting model that incorporates the static and dynamic correlations between temporal variation and spatial diversity, in which the dynamic component accepts and produces dynamic graph based on changes in real-time traffic conditions. The generated dynamic graphs could improve the ability of the potential spatial features mined by the model. At the same time, the comprehensive spatial correlation and global information among different areas of the city should also be captured by combining the static road network information.

With this end in view, we propose a travel demand prediction model named the hybrid dynamic graph convolutional network (HDGCN), fusing both the static and dynamic graphs to reflect travel demand variation over time and space. Inspired by the dynamic time programming (DTW) algorithm [21], we constructed a traffic flow similarity graph and then generated the dynamic graphs sequence on time series. We employed dynamic graph convolution module to extract the spatial features of the generated graph sequences. It is worth noting that we adopted the structure of recurrent networks to improve the effectiveness of dynamic node feature message passing in the dynamic graph convolution module. For static graphs, we used a standard GCN to extract the spatial features. Afterward, we integrated the static and dynamic features with a 2D convolution operation and used the GRU to extract the temporal features. Finally, we conducted forecasting and comparative experiments based on a real-world travel demand dataset from the New York City Taxi and Limousine Commission (TLC). The numerical experiment results show that HDGCN outperforms the benchmark models such as ARIMA and GRU.

To summarize, the main contributions of this paper are as follows:

1. We proposed a new dynamic graph generation method to reflect the real-time travel demand conditions which could generate dynamic graphs sequence on the time series. The dynamic graph convolution module could effectively capture the spatial features possessed by dynamic graphs thus to reduce prediction errors.
2. We designed a spatial-temporal prediction network incorporating static area graphs and traffic flow similarity graphs. This deep learning network combines GCN and GRU, in which the former is used to learn the spatial features, and the latter is used to learn the temporal features of the traffic network.
3. We combined the real-world travel demand data with demonstrating the validity of the proposed model. In the experiments, HDGCN focuses on mining the spatial-temporal properties of historical demand data. The results indicate that HDGCN has great potential in urban travel demand prediction and urban transportation management for its excellent performance in error reduction and temporal stability.

The rest of this paper is organized as follows: Section 2 reviews the related works, and Section 3 introduces the preliminaries. Section 4 provides details of the proposed methodology, and Section 5 presents the analysis of the experimental results. Finally, the conclusions and further research directions are discussed in Section 6.

## 2. Related Works

We reviewed the related work from two main aspects: travel demand forecasting models and dynamic graph neural network theory.

### 2.1. Travel Demand Forecasting Models

Travel demand prediction is essential for optimizing social idle resources and matching travel demand in urban areas [22], which has attracted extensive attention during the past decades. Ahmed et al. [2] used ARIMA to predict the freeway traffic flow and verified the effectiveness of the method through the traffic flow dataset of three cities. Based on multi-source dataset, Stefano et al. [23] considered the correlation between travel demand and traffic flow and used the Kalman filter to predict the travel demand of online car-hailing. Gary et al. [24] used the nearest neighbor nonparametric regression method for short-term traffic flow prediction and verified the effectiveness of the method. Vanajakshi et al. [25] used SVR to predict passengers’ travel time and validated its practicality by using a real travel dataset.

In recent years, with the development of deep learning technology, various deep learning models have been applied to the task of travel demand forecasting. Pang et al. [26] proposed a recurrent neural network model that exploits long-term dependencies between multiple time steps for bus arrival time prediction. Xu et al. [27] proposed a predictive model that uses long short-term memory (LSTM) to train historical taxi demand data to predict future demand changes. However, these time-series-based deep learning models usually ignored the spatial correlation of travel demand among different regions. In recent years, to better explore the spatial correlation of different urban areas, researchers usually divided the modeling methods of traffic network into two types, namely Euclidean space (grid format) [6,28,29,30,31] and non-Euclidean space (graph format) [7,8,15,32,33].

Regarding the research of travel demand prediction using Euclidean space, Liu et al. [6] added a convolutional layer before the LSTM framework and proposed the ConvLSTM model for extracting spatial-temporal information of urban transportation networks. Zhang et al. [28] proposed the ST-ResNet model, which models urban travel demand as a form of raster data, with each grid representing a region, calculates inflows and outflows within the region, and used CNN to mine spatial features. Zhang et al. [29] proposed a multi-task deep learning framework dividing the actual road network map into grid maps, which was fed into a CNN for learning after graph embedding operations. Wu et al. [30] used CNN to mine spatial features and RNN to learn the temporal dynamics in traffic flow, which further improved the accuracy of the model. Yao et al. [31] used LSTM, local CNN, and graph embedding to form three views: the temporal view, spatial view, and semantic view, as a way to mine spatial-temporal features to complete the task of predicting the volume of regional passengers’ travel orders.

With respect to the research of travel demand prediction using non-Euclidean space models, Chai et al. [8] proposed a novel multi-graph convolutional neural network framework to build multiple graphs based on distances and traffic interactions between stations in traffic network and fused these graphs to learn spatial-temporal features through graph convolution and encoding–decoding networks. Li et al. [7] proposed the diffusion convolutional recursive neural network (DCRNN) to model urban road information as a spatial diffusion process and used GRU to capture temporal dynamics. Geng et al. [15] proposed a novel multi-graph convolutional neural network framework to build multiple graphs based on proximity, functional similarity and traffic connectivity between regions in traffic road network, and multi-graph convolution was used to fuse these graphs to learn spatial-temporal features. Yu et al. [32] proposed the spatial-temporal graph convolutional network (STGCN) to improve the prediction performance by incorporating prior knowledge of traffic topology and used GCN and GRU to extract spatial and temporal features, respectively. Zhao et al. [33] proposed the T-GCN and considered the connection relationship between roads to represent the connection relationship of edges.

In the related work of forecasting taxi demand with the combination of multiple models, Chen et al. [34] linked multi-graph and multi-task learning to improve the prediction accuracy by using multiple auxiliary tasks and verified the effectiveness of the model based on a real-world taxi trajectory dataset. Sun et al. [35] formulated travel flow prediction for irregular regions as spatial-temporal graphs and used spatial graph convolution to construct multi-view graph convolutional networks (MVGCN), and the model performance was demonstrated on a dataset of taxis and shared bicycles. Zhang et al. [36] developed a hotspot recommendation model and a demand prediction model by assigning a hotness coefficient to travel hotspot areas for each time period. Yao et al. [31] established a spatial-temporal multi-view neural network model from the temporal, spatial, and environmental context perspectives that affect taxi travel demand.

### 2.2. Dynamic Graph Neural Network

With the widespread application of graph neural networks, the research on graph structure is no longer limited to static graphs but takes turns to study dynamic graph structure with temporal dimensions. The introduction of temporal dimension fundamentally improves the network properties, enabling a more robust representation of network data, and in turn, improving the network learning capabilities. In the dynamic generation of graph sequences, the currently popular approach is to link the prediction of graphs based on the learned dynamic graph representation. Sankar et al. [37] proposed the dynamic self-attention network (DySAT) model using a self-attention mechanism to capture node representations of dynamic graph structures and capture multidimensional dynamics through multi-headed attention. Pareja et al. [38] proposed the evolving graph convolutional network (EvolveGCN) model taking the parameters of each layer of the GCN as the feature variables of the dynamic network by using GRU or LSTM to update the parameters to combine temporal and spatial information. Ma et al. [39] proposed the dynamic GCN model, which is a streaming graph neural network that models dynamic information by using an update component and a propagation component. Xu et al. [40] proposed temporal graph attention (TGAT) layer, which uses a temporal encoding function based on harmonic analysis instead of the position vector in the self-attentive mechanism as a way to handle node classification and link prediction tasks. Trivedi et al. [41] proposed the unsupervised DyRep model, a model that describes the structural information of a graph as node forms based on the temporal evolution process and learns these nodes coded to achieve link prediction.

## 3. Preliminaries

**Definition** **1.** *Geographically adjacent graph: In this paper, we define the traffic network as an undirected graph* $G_{S}=\left(V,E,A_{S}\right)$, *where E is the edge collection of the road network; and* $V=\{v_{1},v_{2},\cdots ,v_{N}\}$*is the set of region nodes, where N is the number of region, and* $A_{s}\in {\mathbb{R}}^{N\times N}$*is the static adjacency matrix, whichis used to represent the connection between nodes*.

**Definition** **2.** *Traffic flow similarity graph: We define the traffic flow similarity graph at moment t as* $G_{D}^{t}=\left(V,E,A_{D}^{t}\right)$ *, where V, E has the same meaning as in the geographically adjacent graph, and* $A_{D}^{t}\in {\mathbb{R}}^{N\times N}$ *is the dynamic adjacency matrix at moment t, which represents a set of similarity of demand patterns on all the regions.*

**Definition** **3.** *Travel demand feature: The travel demand feature* $X^{t}$ *represent the historical travel demand feature for each region in the region at time t. For a traffic network with N region.* $X^{t}=(x_{1}^{t},x_{2}^{t},\cdots ,x_{N}^{t})$ *, where* $x_{j}^{t}$ *is the travel demand of region j at time t.*

**Problem** **1.** *According to the travel demand feature at the time range [t-m-T:t-T] to autonomously generated similarity graph sequence* $G_{D}=\{G_{D}^{t-m},G_{D}^{t+1-m},\cdots ,G_{D}^{t}\}$, *where T is the length of the sequence*.

**Problem** **2.** *Given* $G_{s}$ *, and travel demand feature* $X^{(t-m):t}$ *to learn a function* $f:{\mathbb{R}}^{N\times m}\to {\mathbb{R}}^{N\times 1}$ *that maps historical demands of all regions to the demand in the next time step, which is represented as follows:*

(1) $${\hat{X}}^{t+1}=f(X^{(t-m):t},G_{S},G_{D})\;$$

*where* ${\hat{X}}^{t+1}$ *denotes the predicted travel demand of all regions over the next time step, and the prediction function* f(⋅) *is shared by all regions.*

## 4. Methodology

### 4.1. Framework

**Figure 1:**
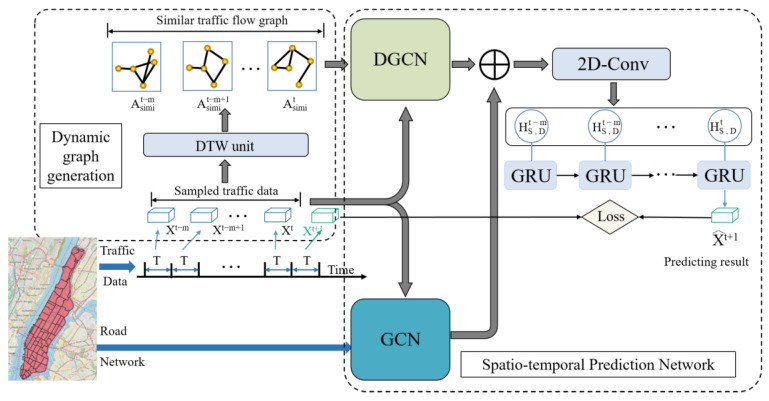
The framework of the HDGCN model.

The framework of HDGCN model is shown in Figure 1, which could be broadly divided into three phases:

Dynamic graph generation: We consider a situation where some urban regions were spatially distant but were similar in travel demand patterns. Therefore, we propose the use of a traffic flow similarity graph to capture this potentially spatial characteristic, where the nodes represent different regions within the city and the edges represent the similarity of the demand patterns for the paired regions, in which the weights of the edges are measured by the dynamic time warping. On this basis, the dynamic graphs sequence based on the original data could be automatically generated according to the time progress.

**Figure 2:**
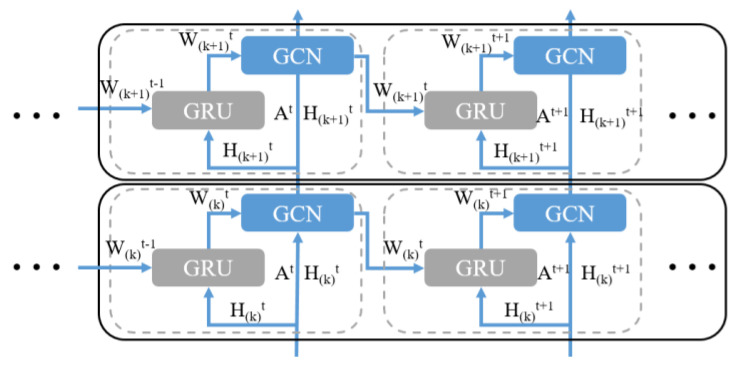
The framework of the dynamic graph convolution module.

Dynamic graph convolution module (DGCN)**:** We use a dynamic graph convolution module, as shown in Figure 2, to extract the similarity of the regional demand models in the dynamic graphs sequence. The dynamic graph convolution module captures the dynamism underlying a dynamic graphs sequence by using a GRU to evolve the GCN parameters.

Spatial-temporal prediction network: We also consider the connected condition of realistic urban areas and extract static urban spatial features in the geographically adjacent graph by GCN. Afterward, a 2D convolution is used to fuse the above two spatial features to obtain the final spatial features. Finally, the spatial feature sequence is used as input to the GRU unit, through which the temporal features could be extracted.

### 4.2. Dynamic Graph Generation

The research using non-Euclidean space to model urban transportation demand usually considered only static spatiality. For example, the neighborhood graph is usually based on the regions’ connectivity, the transportation connectivity graph is generally based on the Euclidean distance, the functional similarity graph is normally based on the different functional regions, etc. Compared to the approaches modeling travel demand using the two-dimensional grid to create urban areas, the methodology adopting non-Euclidean space further considers the difference in topological information among urban regions. However, there are still shortcomings in such a model, and an obvious disadvantage is that it ignores the possible dynamic correlations among regions at different times of the day. For instance, residential districts usually generate high travel demands during the weekday morning and evening peak hours, and regions including parks and suburban attractions generally attract more tourists on weekends than on weekdays. Therefore, we need to consider the dynamic correlation among regions with similar demand patterns, even though these regions are not adjacent to each other.

To reflect this regularity, we propose the concept of a traffic flow similarity graph. The graph is deeply connected because every two regions can interact. If the trend in travel demand between two regions is coincident or similar during the same time period, the link weight between the two regions is given greater weight. Assuming that the sample’s time interval of traffic flow is T, and the traffic flow similarity graph corresponding to time *T* is denoted as $G_{D}^{t}=\left(V,E,A_{D}^{t}\right)$, we use DTW to measure the similarity $\omega_{M,N}^{t}$ between region *M* and region *N* during the time period of (*t − T*, *t*], that is:

(2) $$\omega_{M,N}^{t}=\exp(-\alpha DTW{\left(V_{M},V_{N}\right)}^{t})$$

where α is the parameter that controls the decay rate of distance (in this paper, α = 1), $V_{M}=\{v_{M}^{t-T},v_{M}^{t+1-T},\cdots ,v_{M}^{t}\}$ is the travel demand sequence of region *M* during the time period of (*t* − *T*, *t*], $DTW{\left(V_{M},V_{N}\right)}^{t}$ represents the dynamic time warping distance between the demand patterns of the two regions. The similarity of the changing trend of regions increases with the decrease of $DTW{\left(V_{M},V_{N}\right)}^{t}$. $DTW{\left(V_{M},V_{N}\right)}^{t}$ minimizes the cumulative distance $\gamma (V_{M}^{},V_{N}^{})$ between $V_{M}$ and $V_{N}$ by finding an optimal warping path, that is:

(3) $$DTW(V_{M},V_{N})=\gamma (v_{M}^{t},v_{N}^{t})$$

(4) $$\gamma (v_{M}^{i},v_{N}^{j})=d(v_{M}^{i},v_{N}^{j})+\min\{\begin{array}{c} \gamma (v_{M}^{i-1},v_{N}^{j-1})\\ \gamma (v_{M}^{i-1},v_{N}^{j})\\ \gamma (v_{M}^{i},v_{N}^{j-1}) \end{array}$$

(5) $$d(v_{M}^{i},v_{N}^{j})=\left|v_{M}^{i}-v_{N}^{j}\right|$$

where $d(v_{i}^{M},v_{j}^{N})$ represents the distance measure between two elements.

In practice, we obtain a sequence of dynamic adjacency matrices $A_{D}=\{A_{D}^{1},A_{D}^{2},\cdots ,A_{D}^{t}\}$ sorted chronologically by sequentially calculating the time-series correlations between different regions during different time periods, which is used as the adjacency matrix in the dynamic graphs sequence. It is worth noting that the traffic flow similarity graph generated at time step *T* reflects the similarity of travel demand within time period *T*. Therefore, the consistency of the time step should be maintained in the subsequent spatial-temporal forecasting network.

### 4.3. Dynamic Graph Convolution Module

In order to capture the dynamics behind the traffic flow similarity graph, we add an update mechanism to the standard GCN architecture and use a recurrent model to update the GCN parameters. The recurrent model has various deformation models, such as LSTM and GRN. Considering that nodes includes feature information, our architecture is implemented by using GRU, and the GCN’s weights are treated as hidden states of the recurrent structure. Specifically, at time *t*, the *k*-th layer takes the dynamic adjacency matrix $A_{D}^{t}$ and the node embeds the matrix $H_{k}^{t}$ as the input and uses the weight matrix $W_{k}^{t}$ to update the node embedding matrix $H_{k+1}^{t}$ to as output, that is:

(6) $$ƒ(H_{k}^{t},A_{D}^{t},W_{k}^{t})=\sigma ({\hat{D}}^{t^{-1}}A_{D}^{t}H_{k}^{t}W_{k}^{t})$$

where ƒ(⋅) represents the graph convolution operation and σ(⋅) is the activation function (typically ReLU).

The crucial weight matrix is passed through a GRU to update the hidden state upon time-*t* input to the system.

(7) $$W_{k}^{t}=GRU(H_{k}^{t},W_{k}^{t})=(1-z_{k}^{t})W_{k}^{t-1}+z_{k}^{t}{\hat{w}}_{k}^{t}$$

(8) $$z_{k}^{t}=\sigma (U_{z}H_{k}^{t}+V_{z}W_{k}^{t-1}+B_{z})$$

(9) $$r_{k}^{t}=\sigma (U_{r}H_{k}^{t}+V_{r}W_{k}^{t-1}+B_{r})$$

(10) $${\hat{w}}_{k}^{t}=tanh(U_{w}H_{k}^{t}+V_{w}(r_{k}^{t}W_{k}^{t-1})+B_{w})$$

where $z_{k}^{t}$, $r_{k}^{t}$, and $w_{k}^{t}$ represent the update gate, reset gate, and pre-output at time *t*, respectively, and $U_{*}$, $V_{*}$, and $B_{*}$ represent the weights and biases of the corresponding gate structures, respectively.

In the dynamic graph convolution module, GCN aggregates the node’s neighborhood information by using bottom-up approach, while the GRU updates the weight parameters from the left to the right. As a result, the dynamic graph convolution module can dynamically obtain the hidden information of the nodes.

### 4.4. Spatio-Temporal Prediction Network

To further improve the prediction accuracy in different urban regions, we propose the HDGCN to fully extract the spatial-temporal features. The HDGCN uses the standard GCN to extract the static properties of geographically adjacent graphs and the dynamic graph convolution module extracts the dynamic spatial properties of dynamic graphs’ sequence. Based on the obtained spatial feature sequence, the GRU is used to extract the temporal features and achieve forecasts of future demand requirements for each region within the city.

In spatial feature extraction, the static graphs and the dynamic traffic flow similarity graphs reflect the region correlations from distinct perspectives. Therefore, on the one hand, we use GCN to extract the static spatial correlation of the geographically adjacent graph $G_{S}=\left(V,E,A_{S}\right)$, where the adjacency matrix $A_{S}$ is defined based on the connectivity of neighboring regions. This method determines whether the region is connected with another or not, which is as follows:

(11) $$A_{i,j}=\{\begin{array}{l} 1,\;\mathrm{region}\;i\;\mathrm{and}\;j\;\mathrm{are}\;\mathrm{adjacent}\;\\ 0,\;\mathrm{otherwise} \end{array}$$

On the other hand, we use the dynamic graph convolution module to extract the dynamic spatial correlations of the dynamic graphs’ sequence produced in Section 4.3. After being processed by the former two convolution modules, two kinds of spatial feature matrices are obtained: (1) $H_{S}$ for the static space feature and (2) $H_{D}$ for the dynamic space feature. To combine the two kinds of features, $H_{S}$ and $H_{D}$ are concatenated to form a new matrix, $H_{S,D}$. After that, we use a 2D convolution with a convolution kernel size of 2 × 1 to perform a convolution operation on $H_{S,D}$ and finally output a mixed spatial feature, $H_{all}$.

After extracting the spatial features, we also need to extract the long-term trends and cyclical features of regional travel demand changes to achieve accurate travel demand prediction. Here we add the GRU unit to our spatial-temporal prediction network. The purpose of using the GRU unit to update the weight parameters of the GCN according to the time dimension is to capture the similarities of regional travel demand thus to enhance the performance of spatial feature extraction. The input of the GRU unit is the spatial feature sequence $H_{all}=[h_{all}^{t-T},\cdots ,h_{all}^{t-1},h_{all}^{t}]$ within a certain period of time, and the output is the prediction result ${\hat{X}}^{t+1}$ in the next time step.

When training the model, the mean square error (MSE) is employed as a loss function to calculate the difference between the predicted travel demand ${\hat{Y}}_{t}$ and the real travel demand $Y_{t}$. The goal of model training is to minimize the loss. The calculation method is as follows.

(12) $$loss(Y_{t},{\hat{Y}}_{t})={\left(Y_{t}-{\hat{Y}}_{t}\right)}^{2},\;Y_{t},{\hat{Y}}_{t}\in R^{N\times t}$$

## 5. Experiments

### 5.1. Dataset Description

The dataset used in the experiments contains the real-world and open-source taxi data of Manhattan from the Taxi and Limousine Commission (TLC) of New York City. There are three kinds of taxis on the platform: yellow taxis, green taxis, and For-Hire Vehicles (FHVs). The dataset consists of 12,179,185 yellow taxi orders in the Manhattan district, New York City, from 1 January to 30 June 2021. For each taxi, the global positioning system (GPS) device records 18 data attributes such as VendorID, pickup_datetime, PuLocationID, trip distance, etc. We select several fields for cleaning and prediction, and others are abandoned.

**Figure 3:**
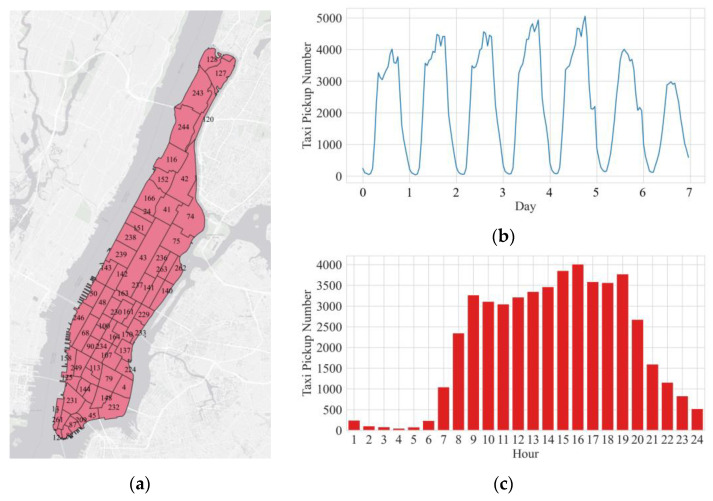
(**a**) The division of 63 regions in the research area (Number from NTAs); (**b**) the hourly taxi demand of one week (8 to 14 March 2021); (**c**) the hourly taxi demand of one day (8 March 2021).

Figure 3 depicts the delineation and basic statistical characteristics of the research area, in which Figure 3a represents the current status that the Manhattan district which is partitioned into 63 regions or zones based on the NYC Department of City Planning’s Neighborhood Tabulation Areas (NTAs), Figure 3b represents the hourly taxi demand of one week, and Figure 3c represents the hourly taxi demand of one day.

### 5.2. Experimental Settings

We set the time interval for the aggregation of travel demand to be 15 min. Therefore, each node contains 96 data points per day. We use the data collected from 27 May to 16 June of 2021 as the validation dataset, and the data collected from 17 June to 30 June of 2021 as the test dataset. The rest of the data is used as the training dataset. In the experiment, we set the time step *T* to be 6, which is crucial for the generation of dynamic graphs as the dynamic graphs’ sequence is generated by using the demand time series within 1.5 h from the current moment as the demand pattern. We use a two-layer dynamic graph convolution module to extract dynamically spatial features, and use a single-layer GCN model to extract statically spatial features. The number of layers of the GRU is set to be 1, and the hidden units are set to be 48. The input length of the GRU unit is consistent with the time step of the dynamic graph, and the input sequence is the mixed spatial feature in the past 1.5 h. In the experiment, we select the optimal batch size to be 64, the learning rate is set to be 0.0001, and the number of epochs is set to be 100. To make a fair evaluation, we use the same dataset for the model training, validation, and testing across all experimental models. We implement all the methods in Python and use PyTorch for the neural network-based approaches.

Three commonly used metrics were used to evaluate the prediction performance of our model: mean absolute error (MAE), mean absolute percentage error (MAPE), and root mean square error (RMSE), which are defined as follows:

(13) $$\mathrm{MAE}=\frac{1}{N}\sum_{i=1}^{N}\left|y_{i}-{\hat{y}}_{i}\right|$$

(14) $$\mathrm{MAPE}=\frac{1}{N}\sum_{i=1}^{N}\frac{\left|y_{i}-{\hat{y}}_{i}\right|}{y_{i}}$$

(15) $$\mathrm{RMSE}=\sqrt{\frac{1}{N}\sum_{i=1}^{N}{\left(y_{i}-{\hat{y}}_{i}\right)}^{2}}$$

where *N* is the number of prediction results ${\hat{y}}_{i}$ and $y_{i}$ is the ground truth value.

### 5.3. Baselines

We compare the HDGCN with the following 10 baselines:

- ARIMA, which is a popular prediction model using a time series forecasting method that combines autoregressive and moving average.
- SVR, which is a classic machine learning model for time series analysis that uses linear support vector machines for regression.
- Extreme Gradient Boosting (XGBoost), which is an efficient, flexible, and portable integrated learning algorithm that comprises many CART trees.
- Multiple layer perceptron (MLP), which is a common feedforward neural network with strong self-adaptation and self-learning capabilities. MLP can solve nonlinear and high-dimensional problems through special activation functions.
- GRU, which is an extension of RNN with good performance in time series modeling. We forecast future demand in each region by establishing a GRU for each region, and its settings are consistent with the GRU settings in DHGNN.
- GCN, which is essentially a feature extractor that takes graph data as the object. In this paper, we build a two-layer graph convolutional neural network with geographically adjacent graph as the input of GCN, and use the fully connected layer to predict the demand of all regions.
- DCRNN, which designs a diffusion convolutional neural network to model roads, which combines GRU and bidirectional diffusion convolution. It is a data-driven prediction framework.
- T-GCN, which integrates GCN and GRU. GCN is used to capture spatial dependencies, and GRU is used to extract temporal dependencies.
- STGCN, which applies the full convolutional structure to build the model and is an integrative framework of graph convolution network and convolutional sequence.
- DySAT, which is a dynamic graph convolution algorithm based on discrete networks. It aggregates the node features of each snapshot through GCN and then aggregates the temporal features of each node in each snapshot by self-attention.

For the ARIMA model, the input is the demand intensity sequence in a single region after sequence stabilization. For SVR, XGBoost, MLP, and GRU, the input that the model accepts each time is a feature vector of a single region in a time interval. For GCN, DCRNN, T-GCN, and STGCN, the input consists of an adjacency matrix describing the spatial relationships between regions (geographically adjacent graph), and the feature matrix composed of the feature vectors of all regions in the same time interval. For DySAT, the input received each time includes the dynamic adjacency matrix used to describe the dynamic demand similarity between regions (traffic flow similarity graph) and the feature matrix composed of feature vectors of all regions in the same time interval.

### 5.4. Experiment Results and Analysis

#### 5.4.1. Modeling Performance Compared with the Baseline Models

Table 1 reveals the performance of the proposed prediction network compared with the 10 baseline models as mentioned above. The proposed HDGCN model achieves the best prediction results with the minimum values of MAE (3.95), MAPE (17.60%), and RMSE (6.21). Compared with the baseline models, the MAE, MAPE, and MAPE of the proposed HDGCN model at least decrease by 10.43%, 12.70%, and 13.03%, respectively, largely due to its wonderful consideration of both the statically spatial proximity and dynamically spatial variation of travel demand. The traditional time series methods, such as ARIMA, SVR, and XGBosst, only consider temporal correlation but ignore the spatial correlation, thus resulting in poor performance in travel demand forecasting with long-term non-stationary characteristics. While the performance of MLP and GRU is slightly better in eliminating errors regard than ARIMA and XGBosst, considering only temporal features and ignoring spatial features is clearly not as comprehensive as extracting both temporal and spatial features. Both the GCN and DySAT consider only spatial features but ignore the impact of temporal dependence, thus producing relatively large errors. DySAT considers travel demand dynamics between different regions by capturing dynamic spatial dependencies at each time step, with obviously improved performance, which suggests the effectiveness of dynamic graph model in improving prediction accuracy. The DCRNN, STGCN, and T-GCN are the most state-of-the-art baseline methods that comprehensively consider spatial-temporal features, but their graph modeling only considers the geographical proximity information without considering the dynamic changes in travel demand patterns among regions.

#### 5.4.2. Modeling Performance for Different Time Periods and Urban Areas

**Figure 4:**
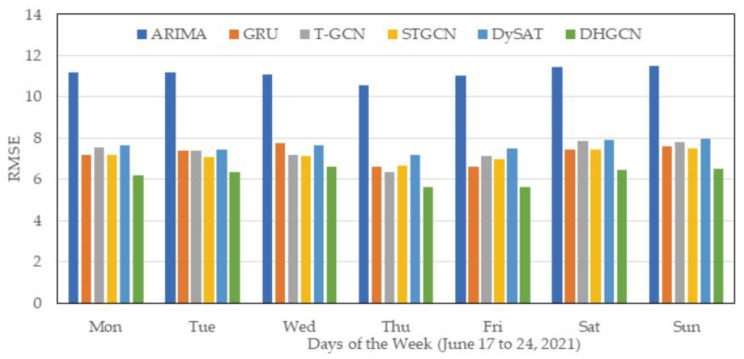
Modeling performance for days of the week (17 to 24 June 2021).

Figure 4 exhibits the modeling performance of different methods for 7 days of the week (17 to 24 June 2021). Due to space limitations, we compared the modeling performance of HDGCN in MAPEs with that of ARIMA, GRU, T-GCN, STGCN, and DySAT as the benchmarks. Obviously, compared with the baseline models, the proposed HDGCN model has the best performance in reducing the RMSEs for all 7 days of the week. Moreover, we observe that the estimating errors for weekends are generally higher than for weekdays. For instance, the average RMSE of HDGCN estimated for 23 and 24 June (Sat. and Sun.) is 6.50, compared with an average value of 6.10 for 17 to 22 June. The results indicate that it is relatively more challenging to predict travel demand on weekends than on weekdays. Two potential reasons may lead to this result: (1) the travel demand base on weekends is generally smaller than that on weekdays, and (2) people have more regular travel needs on weekdays (such as working and schooling) than on weekends (such as shopping and others).

**Figure 5:**
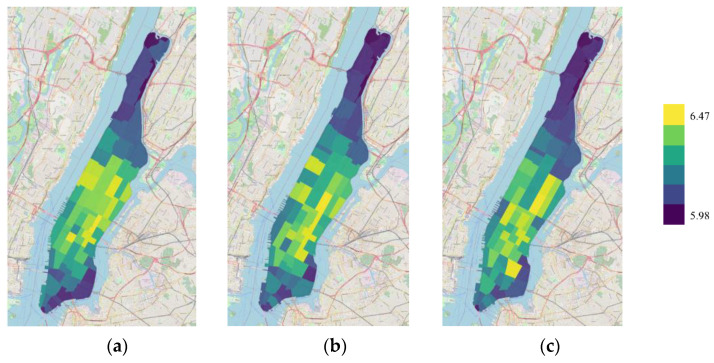
Heat maps of spatial distribution of HDGCN’s RMSE at different time periods: (**a**) 9:00; (**b**) 18:00; (**c**) 22:00.

Figure 5 presents the prediction performance of HDGCN in the regions of Manhattan. In order to have a more intuitive understanding of the modeling performance in different regions during the main time periods, we use the heat maps as shown in Figure 5a–c to present the RMSE distributions in Manhattan at the hours of 9:00, 18:00, and 22:00, respectively. It can be seen that the prediction error of the region-based approach is smaller in sparsely populated regions. The reason may be that when the travel demand is small, a large number of zero elements appear in the time series between regions, and the link weights in the dynamic adjacency matrix calculated in this way are less different, which affects the generation of dynamic graphs and the extraction of spatial features. Moreover, within the same area, the error is higher in the morning peak (9:00) and evening peak (18:00) than in leisure time (22:00).

**Figure 6:**
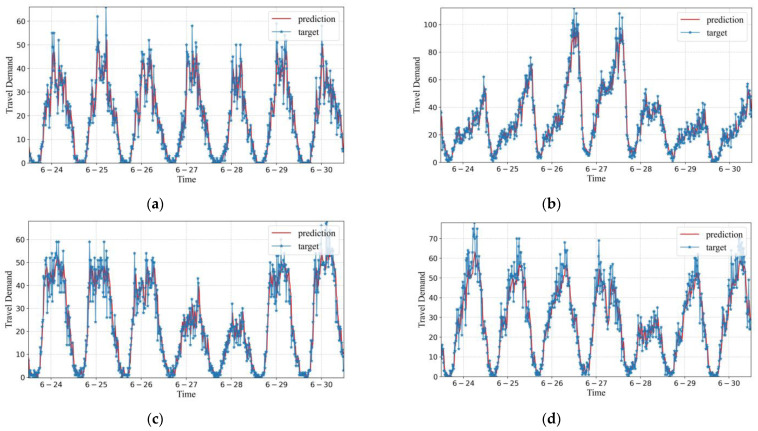
Visualization of the prediction results and actual travel demand based on HDGCN model in different regions within the study area: (**a**) VendorID 43; (**b**) VendorID 73; (**c**) VendorID 140; (**d**) VendorID 233.

Figure 6 shows the predicting results of the HDGCN compared with the actual value of travel demand in the four areas of VendorID 43, VendorID 73, VendorID 140, and VendorID 233 as having been shown in Figure 1. While the distributions of travel demand in the four areas express obviously different demand patterns and intensities, the HDGCN modeling results have high fitting degree and applicability, indicating that the proposed model can effectively learn the changing laws from the regional travel demand data with different demand intensities and patterns.

Table 2 summarizes the HDGCN modeling results of different travel days (weekdays and weekends) and different times of weekdays (9:00 and 14:00) for the four areas of VendorID 43, VendorID 73, VendorID 140, and VendorID 233. While the predicting results of the HDGCN generally fit well with the actual value, it should be noted that the goodness of fit of modeling results for weekends is generally lower than that for weekdays (consistent with the foregoing modeling results), and the goodness of fit of modeling results for the peak hour 9:00 AM are generally lower than that for the non-peak hour 14:00. Consistent with the modeling results as shown in Figure 5, other factors affecting the diversity of travel demand, particularly during peak hours, should be further studied in the future. In other words, if the HDGCN model is combined with other disaggregate factors, such as parameters reflecting daily time periods, regionally functional facilities, and road conditions among areas, the modeling performance could be further improved.

#### 5.4.3. Modeling Performance for Different Time Length

**Figure 7:**
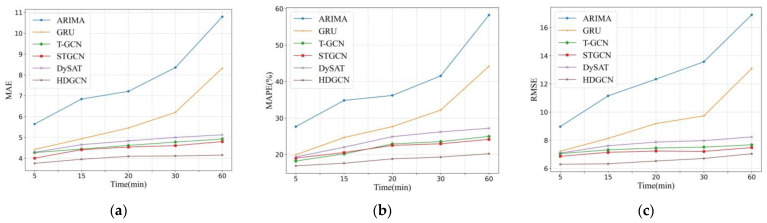
Performance comparison of different model at different length of prediction periods: (**a**) MAE; (**b**) MAPE; (**c**) RMSE.

Figure 7 shows the travel demand forecasting performance of the HDGCN for the next 5 min, 15 min, 30 min, and 60 min, taking ARIMA, GRU, T-GCN, STGCN, and DySAT as the benchmarks for comparison. In which, Figure 7a–c represents MAE, MAPE, and RMSE, respectively. It can be seen that with the increase in time duration, the errors of modeling results show an upward trend, especially the modeling results of ARIMA and GRU. However, the predicting errors of HDGCN rise at the slowest rate than all the other models, indicating that the HDGCN modeling results are robust over time.

### 5.5. Ablation Experiment

In this section, we further evaluate the effects of key components that may contribute to improve the performance of the HDGCN model. Three variants of the HDGCN model are defined as follows:

1. HDGCN-AD: HDGCN simply connects the GCN and GRU, without mixing the dynamic adjacency matrix.
2. HDGCN-AS: HDGCN, without mixing the geographically adjacent graph convolution.
3. HDGCN-GRU: HDGCN only considers the spatial module and predicts through a simple connected layer, without mixing the time series module.

**Figure 8:**
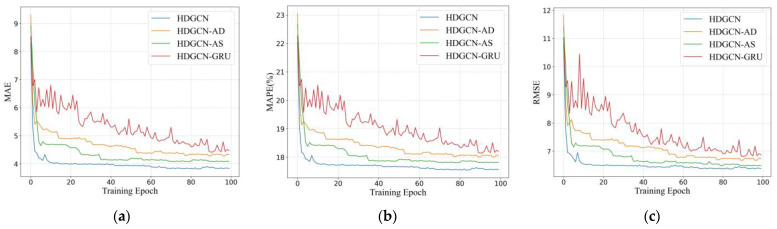
Performance comparison of the HDGCN model and its variants at different epochs on the validation set: (**a**) MAE; (**b**) MAPE; (**c**) RMSE.

Figure 8 shows the modeling results of MAE (Figure 8a), MAPE (Figure 8b), and RMSE (Figure 8c) with the number of training epochs. The results show that MAE, MAPE, and RMSE of all models decrease obviously with the number of training epochs increasing from 0 to 20 and then tend to be relatively stable with the number of training epochs increasing from 20 to 100. Comparing HDGCN, HDGCN-AD, and HDGCN-AS, it can be seen that considering spatial features is important for predicting regional travel demand. Concurrently, applications of dynamic graphs are effective in reducing errors, which can effectively improve the accuracy of travel demand forecasting. Compared with HDGCN-GRU, which only considers a spatial correlation, HDGCN expresses an obvious improvement. We can see that it is unwise to consider only the features of spatial correlation without considering the time dimension, which will generate an obvious shake in the training process.

## 6. Conclusions and Future Work

The paper set out to propose an improved model, i.e., HDGCN, related to urban travel demand prediction. This model takes into account regional connectivity and travel pattern correlations between regions, collaborating dynamic graphs, and static graphs to capture spatial correlations more comprehensively and was designed based on the similarity of travel demand between different regions and generated a dynamic graph sequence in the time dimension. Meanwhile, for the dynamic graph sequences, we proposed a new dynamic graph convolution method to extract spatial features, which can adaptively learn the parameters of the adjacency matrix and update the parameters of the GCN according to the dynamic information of each time step. We extracted the temporal features through GRU and trained deep learning models to improve the predicting accuracy of travel demand.

We evaluated the effectiveness and robustness of the proposed model by using the real-world and open-source taxi data of Manhattan, New York City. The experimental results demonstrate that the proposed model outperforms the mainstream machine learning and deep learning methods, which could be used for the real-time, accurate, and efficient travel demand prediction of urban taxis and other transportation systems. In the end, although this paper constructs a sequence of dynamic graphs in urban travel and integrates them with an urban static graph, some limitations of the current study for further improvement are given for reference. First, we used the discrete network to generate the dynamic graph, but in future research, the continuous network for dynamic graph generation could be considered. Second, due to space constraints, we did not discuss the modeling results over time for all the regions in Manhattan in detail. Finally, many other disaggregate factors such as personal characteristics that may affect travel demand could be considered in future research.

## Figures and Tables

**Table 1:** Performance comparison of different methods.

Methods	MAE	MAPE (%)	RMSE
ARIMA	6.84	34.82	11.15
SVR	5.09	23.70	8.37
XGBoost	5.58	27.93	9.82
MLP	5.18	25.43	8.25
GRU	4.93	24.65	8.13
GCN	5.65	30.14	8.98
DCRNN	4.57	21.27	7.43
T-GCN	4.44	20.16	7.32
STGCN	4.41	20.53	7.14
DySAT	4.65	21.99	7.61
DHGCN	3.95	17.60	6.21

**Table 2:** RMSE performance of HDGCN modeling results during different time periods.

Observation Area	Different Travel Days		Different Times of Weekdays	
	Weekdays	Weekends	9:00	14:00
VendorID 43	5.82	6.08	4.87	4.93
VendorID 73	6.29	8.34	4.8	3.33
VendorID 140	5.54	5.88	7.42	5.56
VendorID 233	6.17	6.53	6.19	5.56

## Data Availability

The data will be released on https://github.com/MIKe-eve/HDGCN (accessed on 5 July 2022).
